# Small molecule compounds that induce cellular senescence

**DOI:** 10.1111/acel.12518

**Published:** 2016-09-14

**Authors:** Nadezhda V. Petrova, Artem K. Velichko, Sergey V. Razin, Omar L. Kantidze

**Affiliations:** ^1^ Institute of Gene Biology RAS 34/5 Vavilova Street 119334 Moscow Russia; ^2^ Department of Molecular Biology Lomonosov Moscow State University 119991 Moscow Russia; ^3^ LIA 1066 French‐Russian Joint Cancer Research Laboratory 94805 Villejuif France

**Keywords:** cellular senescence, cell stress, DNA damage, DNA replication stress, epigenetic modifiers, aging

## Abstract

To date, dozens of stress‐induced cellular senescence phenotypes have been reported. These cellular senescence states may differ substantially from each other, as well as from replicative senescence through the presence of specific senescence features. Here, we attempted to catalog virtually all of the cellular senescence‐like states that can be induced by low molecular weight compounds. We summarized biological markers, molecular pathways involved in senescence establishment, and specific traits of cellular senescence states induced by more than fifty small molecule compounds.

Cellular senescence is a stable arrest of the cell cycle and is characterized by complex phenotypic changes. It was first described in studies of human fibroblasts that ceased proliferation following an extended cultivation (Hayflick & Moorhead, 1961; Hayflick, 1965). Discovered by Hayflick and Moorhead, senescence in normal human cells was shown to depend on telomere dysfunction originating mainly from replication‐associated telomere shortening (Harley *et al*., 1990; Allsopp, 1996; Bodnar *et al*., 1998). This type of senescence is also known as replicative senescence and is the prototypical cellular senescence state. Other forms of senescence (i.e., not linked to proliferation‐dependent telomere shortening) include a variety of prematurely developed cellular senescence phenotypes, similar but not identical to replicative senescence. Many proliferative cell types can undergo so‐called stress‐induced premature senescence (SIPS) upon exposure to subcytotoxic stresses (UV, γ‐irradiation, H_2_O_2_, hyperoxia, etc.) (Toussaint *et al*., 2000, 2002). Oncogene‐induced senescence (OIS) represents another complex senescence phenotype that depends on activation and/or overexpression of oncogenes (Serrano *et al*., 1997; Bianchi‐Smiraglia & Nikiforov, 2012). The mechanism of OIS involves DNA damage that may be a result of DNA hyper‐replication (Di Micco *et al*., 2006), replication fork reversal (Neelsen *et al*., 2013), depletion of nucleotide pools (Mannava *et al*., 2013), and/or increased levels of reactive oxygen species (ROS) (Lee *et al*., 1999). Conceptually and mechanistically, OIS is closely related to tumor‐suppressor loss‐induced senescence (Chen *et al*., 2005; Di Mitri & Alimonti, 2016). Cell‐to‐cell fusion‐induced senescence can also be considered a premature senescence subtype (Chuprin *et al*., 2013; Burton & Faragher, 2015). The distinctive phenotypic changes typical of various types of cellular senescence are cell enlargement and flattening, senescence‐associated β‐galactosidase activity (SA‐β‐gal), formation of senescence‐associated heterochromatin foci (SAHF), persistent DNA damage response (DDR), and senescence‐associated secretory phenotype (SASP). However, these and several other facultative features of cellular senescence that manifest in each particular case of cell cycle arrest greatly depend on the senescence‐inducing stimulus and the cell type (Campisi, 2013; Salama *et al*., 2014).

The contribution of cellular senescence to organismal aging is a question of ongoing research (van Deursen, 2014). However, strong evidence for this connection has been reported recently. Specifically, it was shown that clearance of age‐accumulated p16^INK4A^‐positive senescent cells in mice could extend their healthy lifespan (Baker *et al*., 2011, 2016). Several chemical compounds that specifically target senescent cells have been identified in the last 2 years (so‐called senolytic drugs) (Xu *et al*., 2015b; Zhu *et al*., 2015a,b). It was shown that clearance of senescent cells by such drugs may alleviate age‐related vasomotor dysfunction and frailty, enhance adipogenesis, rejuvenate haematopoietic stem cells after total‐body irradiation, and, generally, extend lifespan (Xu *et al*., 2015a; Zhu *et al*., 2015b; Roos *et al*., 2016). Furthermore, these studies confirm the known pathological impact of cellular senescence, exemplified by cellular dysfunction, impairment of tissue regeneration, detrimental effects on tissue microenvironment, etc. (Burton & Krizhanovsky, 2014). It is evident that along with its detrimental effects, cellular senescence has clearly defined beneficial physiological functions. For instance, it has been shown recently that cellular senescence plays a role in the differentiation of megakaryocytes (Besancenot *et al*., 2010), the maturation of the placenta (Chuprin *et al*., 2013), the restriction of fibrosis (Krizhanovsky *et al*., 2008; Jun & Lau, 2010; Zhu *et al*., 2013), tissue repair (Demaria *et al*., 2014), and embryonic development (Nacher *et al*., 2006; Munoz‐Espin *et al*., 2013; Storer *et al*., 2013). The role of cellular senescence in cancer prevention is well documented (Burton & Krizhanovsky, 2014; Munoz‐Espin & Serrano, 2014).

It is generally agreed in the field that the most important features of cellular senescence are SASP and resistance to apoptosis (Munoz‐Espin & Serrano, 2014; Burton & Faragher, 2015). SASP stimulates immune system‐dependent elimination of unwanted precancerous cells or specific embryonic cells that undergo senescence. Notably, cellular senescence may serve as an alternative to apoptosis in embryonic development as well as in cancer prevention (Childs *et al*., 2014). It has been shown that failure to undergo senescence triggers apoptosis in a compensatory manner to eliminate transient structures during development (Munoz‐Espin *et al*., 2013; Storer *et al*., 2013). Therefore, it may be reasonable to consider some of the cellular senescence states (e.g., SIPS), along with apoptosis, autophagy, necrosis, etc., in terms of the cell stress response rather than aging. However, it is unclear whether or not the cellular senescence that is widely implicated in normal aging, chronic diseases, tumor suppression, tumorigenesis, cell differentiation, and embryogenesis represents a single physiological cellular state.

To date, dozens of stress‐induced cellular senescence phenotypes have been reported. These senescence states may differ substantially from each other, as well as from replicative senescence, through the presence of specific senescence features. Additionally, it has been reported that some stress‐induced senescence states can be overcome, thus challenging the dogma that cellular senescence is an irreversible form of growth arrest (Romanov *et al*., 2001; Beausejour *et al*., 2003). Such caveats can lead to confusion regarding the terminology of stress‐induced cellular senescence states; it is not clear whether senescence‐like growth arrest (and variations thereof) resembles ‘true’ cellular senescence. The indispensable characteristics of this ‘true’ cellular senescence are also elusive. It can be argued that SASP (arising along with morphological changes and SA‐β‐gal) may be the most important physiologically relevant feature of cellular senescence; however, SASP has not been studied in most cases of stress‐induced senescence. Here, we attempt to catalog virtually all of the cellular senescence‐like states that can be induced by low molecular weight compounds (Table 1). We summarize the biological markers, molecular pathways involved in senescence establishment, and specific traits of cellular senescence states induced by small compounds, as well as the treatment conditions used. In total, we analyzed more than 50 chemical inducers of cellular senescence and senescence‐like states. These chemical compounds can be functionally classified into eight groups: (1) DNA replication stress inducers (aphidicolin, hydroxyurea, thymidine, bromodeoxyuridine, difluorodeoxycytidine, cyclopentenyl cytosine); (2) DNA‐damaging agents, including (2a) DNA topoisomerase inhibitors (doxorubicin, etoposide, daunorubicin, mitoxantrone, camptothecin), (2b) DNA cross‐linkers (cisplatin, mitomycin C, busulfan, cyclophosphamide, diaziquone), and (2c) drugs with complex effects (actinomycin D, bleomycin, temozolomide); (3) epigenetic modifiers that inhibit DNA methyltransferases (5‐aza‐2′‐deoxycytidine), histone deacetylases (sodium butyrate, trichostatin A, MS‐275, SAHA, LBH589, phenylbutyric acid, valproic acid), histone acetyltransferases (curcumin, C646), and histone methyltransferases (BRD4770); (4) inhibitors of telomerase activity (SYUIQ‐5, BMVC4, pyridostatin, compound 115405, perylene and indole derivatives, harmine, BIBR1532, azidothymidine); (5) inhibitors of cyclin‐dependent kinases (palbociclib, roscovitine, ribociclib); (6) activators of p53 (nutlin 3a, FL118); (7) activators of protein kinase C (TPA/PMA, PEP005, PEP008); and (8) reactive oxygen species (ROS) inducers (hydrogen peroxide, tert‐butyl hydroperoxide, phenyl‐2‐pyridyl ketoxime, phenylaminonaphthoquinones, paraquat).

**Table 1:** Low molecular weight compounds that induce cellular senescence

Small compounds/Mechanism of action	Cell line	Cellular senescence state (as described by authors)	Senescence markers documented	Signaling pathways involved	Cell cycle phase of the stable growth arrest	Triggering conditions/Other notes (if any)	References
(1) DNA replication stress inducers							
aphidicolin Inhibitor of DNA polymerase α	HFF^a^ REF52	Senescence‐like arrest	Growth arrest γH2A.X foci	p53‐p21↑^▼^ c p‐Rb↓		150–200 nm for ~10 days	Marusyk *et al*. (2007)
	MCF10 MCF7	Prolonged S‐phase senescence‐like arrest	Large flattened cells with increased nuclear size (CS^b^‐like morphology) SA‐β‐Gal γH2A.X foci increased H3K9‐trimethylation	p21↑ p‐Rb↓	S	1 μg mL^−1^ for 4 days reversible state of S‐phase arrest RPA foci	Maya‐Mendoza *et al*. (2014)
hydroxyurea Ribonucleotide reductase inhibitor	HFF	Senescence‐like arrest	Growth inhibition CS‐like morphology SA‐β‐Gal	p53‐p21↑		400–800 μm for ~3 weeks	Yeo *et al*. (2000)
	McA‐RH7777	Senescence‐like arrest	Growth inhibition	p21↑	G1	200–400 μm for 4 days	Hong *et al*. (2004)
	HFF REF52 MCF10A	Senescence‐like arrest	Growth inhibition CS‐like morphology SA‐β‐Gal γH2A.X foci	p53‐p21↑^▼^ p‐p53Ser15↑ p‐p53Ser20↑ p16‐independent		100–150 μm for ~10 days	Marusyk *et al*. (2007)
	K562	Senescence‐like arrest	SA‐β‐Gal	p16↑ p21↑ p27↑		50–600 μm for up to 14 days	Park *et al*. (2000)
thymidine Excess of thymidine inhibits DNA replication by reducing the amount of dCTP synthesized	HeLa TIG‐7	Premature senescence	Growth inhibition CS‐like morphology SA‐β‐gal	ERK1 and/or ERK2↑^▼^		1.5 mm for 7–10 days	Sumikawa *et al*. (2005), Kobayashi *et al*. (2012)
bromodeoxyuridine Suppresses DNA replication	HeLa S3 TIG‐7	Senescence‐like arrest		p21↑		50 μm for 4 days	Eriko *et al*. (1999), Suzuki *et al*. (2001)
	A549	Premature senescence	Growth inhibition CS‐like morphology SA‐β‐gal	p53‐p21↑ p‐Rb↓ p27↑ p57↑	S G2	200 μm for 7 days Chk1Ser345p↑ Chk2Thr68p↑	Masterson & O'Dea (2007)
	HeLa A549	Premature senescence	Growth inhibition CS‐like morphology γH2AX foci SASP	p21↑ DDR (ATM)^▼^	G1	100 μm for 48 h elevated ROS levels p53 activation in A549 cells	Nair *et al*. (2015)
2′,2′‐difluorodeoxycytidine (gemcitabine) Inhibits ribonucleotide reductase Inhibits CTP synthetase	AsPC1 PANC‐1	Premature senescence	Growth inhibition CS‐like morphology SA‐β‐Gal	p21↑	Sub‐G1	100 nm for 4 days	Modrak *et al*. (2009)
cyclopentenyl cytosine Inhibits CTP synthetase	MCF‐7	Premature senescence	Growth inhibition CS‐like morphology SA‐β‐Gal	p53^▼^ p53, p21↑	G2 S	0.125–1 μm for 5 days	Huang *et al*. (2011)
(2) DNA‐damaging agents							
(2a) DNA topoisomerases inhibitors							
doxorubicin DNA intercalator Induces DSBs by poisoning DNA topoisomerase II Induces nucleosome eviction	11 cell lines derived from different types of human solid tumors	Senescence‐like phenotype	Growth inhibition CS‐like morphology SA‐β‐gal	Can be dependent or not on p53 activation		20–50 nm for 3–6 days	Chang *et al*. (1999a)
	HCT116	Senescence‐like phenotype	CS‐like morphology SA‐β‐gal	p53‐p21^▼^ p21↑	G2 phase	50–100 nm for 1–4 days treatment led to the appearance of a substantial fraction of polyploid nuclei in p53^−/−^ and p21^−/−^ lines	Chang *et al*. (1999b)
	MCF7	Premature senescence	CS‐like morphology SA‐β‐gal	p53^▼^ p53, p21↑		1 μm for 2 h	Elmore *et al*. (2002)
	HCT116	Senescence‐like phenotype	CS‐like morphology SA‐β‐gal	p53, p21↑	G2	0.1 μM for 24 h	Sliwinska *et al*. (2009)
	Neonatal rat cardiomyocytes H9c2	Premature senescence	CS‐like morphology SA‐β‐gal	p53↑^▼^ p‐p38↑ p‐JNK↑ p‐ERK↑ MAPK (p‐38 and JNK)^▼^	S	0.1 μm for 3 h	Spallarossa *et al*. (2009)
	WI38	Premature senescence	CS‐like morphology SA‐β‐gal	mTOR^▼^ p53, p21↑		100 ng mL^−1^ for 1–4 days low p53 levels during prolonged cell cycle arrest lead to senescence, while high levels of p53—to either quiescence or cell death	Leontieva *et al*. (2010)
	A549	Transient senescence‐like state	CS‐like morphology SA‐β‐gal		G2	50–200 nm for 72 h	Litwiniec *et al*. (2010)
	MMTV‐Wnt1 mice MCF7	Premature senescence	Growth inhibition SA‐β‐gal SASP	p53^▼^, +/− p21^▼^	G1 (p53‐and p21‐dependent), G2 (p21‐independent)	4 mg kg^−1^day^−1^ for 5 days, MCF7 treated with 200 nm for 24 h *in vivo*	Jackson *et al*. (2012)
	Cardiac progenitor cells	Premature senescence	CS‐like morphology SA‐β‐gal γH2AX	p16↑		0.1–1 μm for 24–48 h *in vivo*	Piegari *et al*. (2013)
	DU145 LNCaP PC3	Premature senescence	Growth inhibition CS‐like morphology SA‐β‐Gal	p21↑ p27↑ p‐Rb↓ p53‐independent	G1	10 nm for 1–5 days	Park *et al*. (2006)
etoposide Poison of DNA topoisomerase II Induces DSBs	LS174T A2780 MCF7	Premature senescence	Growth arrest CS‐like morphology SA‐β‐gal		G1	2 μm for 24 h	te Poele *et al*. (2002)
	WI38	Premature senescence	Growth inhibition CS‐like morphology SA‐β‐gal	p53^▼^ p‐p53↑ p21↑	G1	20 μm for 24 h H2AX phosphorylation, peaked around 8 h and completely resolved at 24 h after the treatment	Probin *et al*. (2006)
	A549	Senescence‐like phenotype	Growth inhibition CS‐like morphology SA‐β‐gal SAHF	p21↑	G2	0.75–3 μm for 72 h Polyploid (higher DNA contents (>G2))	Litwiniec *et al*. (2013)
daunorubicin DNA intercalator Poises topoisomerase II	Jurkat	Senescence‐like phenotype	Growth inhibition SA‐β‐Gal	p53‐p21↑	G2	91 nm for 24 h	Mansilla *et al*. (2003)
mitoxantrone Topoisomerase IIβ inhibitor Induces DSBs	Epithelial cells in biopsies from human prostate cancer patients	Premature senescence	SASP	p21↑ p16↑		*in vivo*	Coppe *et al*. (2008)
	A549 WI‐38	Premature senescence	Growth inhibition CS‐like morphology SA‐β‐Gal γ‐H2AX	p21↑ p‐ATM(Ser1981)↑	G1 G2	2 nm for 2–5 days	Zhao *et al*. (2010)
camptothecin and SN‐38 Topoisomerase I poison Induces SSBs	LS174T MCF‐7 A2780 HCT116	Senescence‐like arrest	Growth arrest CS‐like morphology SA‐β‐gal	p53‐p21↑^▼^ p16↑	G1 S G2	6–100 ng mL^−1^ for 24–168 h	te Poele *et al*. (2002)
	HCT116	Premature senescence	Growth inhibition CS‐like morphology SA‐β‐gal	p53‐p21↑^▼^		20 nm for 24–120 h high concentration (250 nm) of camptothecin results in apoptosis	Han *et al*. (2002)
	H1299	Premature senescence	CS‐like morphology SA‐β‐gal	ATM/ATR^▼^	G2	30–60 nm for 2–3 days p53‐, p16‐, p38‐independent	Roberson *et al*. (2005)
	HCT116	Premature senescence	Growth inhibition CS‐like morphology SA‐β‐gal γH2AX	ATM‐Chk2‐p53‐p21^▼^ p‐ATM↑ p‐Chk2↑ p53↑ p21↑		20 nm for 72 h	Zhang *et al*. (2014)
	HeLa	Senescence‐like growth arrest	Growth inhibition CS‐like morphology SA‐β‐gal γH2AX	p21↑	G2	10–100 nm for 1 h	Velichko *et al*. (2015)
(2b) DNA cross‐linkers							
cisplatin DNA‐alkylating agent Induces DNA intrastrand cross‐links	CNE1	Senescence‐like arrest	Growth inhibition SA‐β‐Gal		S G2	0.5 mkg mL^−1^ for 24 h higher doses result in cell death	Wang *et al*. (1998)
	Normal human lung fibroblasts	Premature senescence	Growth inhibition CS‐like morphology	p53↑	G1	10 μm for 24 h	Zhao *et al*. (2004)
	Human non‐small cell lung cancer cells	Senescence‐like arrest	Growth inhibition SA‐β‐gal	p16^▼^	G2	5 μm for 3 days	Fang *et al*. (2007)
	HCT116	Premature senescence	Growth inhibition SA‐β‐Gal γH2AX foci	p53↑		5 μm for 6 h higher concentrations induce apoptosis	Berndtsson *et al*. (2007)
	HepG2	Premature senescence	Growth inhibition CS‐like morphology SA‐β‐Gal	p53‐p21↑		2 μg mL^−1^ for 48 h ROS↑‐dependent	Qu *et al*. (2014)
	CCL23 CAL27 UM‐SCC1 UM‐SCC14A	Premature senescence	SA‐β‐Gal increased secretion of IL‐8	p53↑ p16↑ p‐Rb↓		6 μg mL^−1^ for 4 h	Veena *et al*. (2014)
mitomycin C DNA‐alkylating agent Induces DNA interstrand cross‐links	A549	Premature senescence	Growth inhibition CS‐like morphology SA‐β‐Gal γH2AX	p21↑	G2	0.01–0.02 μg mL^−1^ for 6 days	McKenna *et al*. (2012)
busulfan DNA‐alkylating agent Induces DNA intrastrand cross‐link	Murine bone marrow cells	Premature senescence	Growth inhibition SA‐β‐Gal	p16↑ p19↑		30 μm for 6 h	Meng *et al*. (2003)
	WI38	Premature senescence	Growth inhibition CS‐like morphology SA‐β‐gal	MAPK(p38, ERK)^▼^ p‐p38↑ p‐JNK↑ p‐ERK↑ p21↑ p16↑	G2	7.5–120 μm for 24 h	Probin *et al*., 2006, 2007
cyclophosphamide DNA‐alkylating agent Induces DNA intrastrand and interstrand cross‐links	Lymphoma‐bearing C57BL/6 mice	Premature senescence	SA‐β‐gal	p53↑^▼^ p16↑^▼^		300 mg kg^−1^ day^−1^ for 7 days *in vivo*	Schmitt *et al*. (2002)
	TIG‐7	Premature senescence	Growth inhibition SA‐β‐gal	MAPK (p‐p38, p‐JNK, p‐ERK)↑^▼^ p21↑ p16↑	G1 G2	10 μm for 14 days	Palaniyappan (2009)
diaziquone DNA‐alkylating agent Induces DNA–DNA and DNA–RNA interstrand cross‐links	DU145	Premature senescence	CS‐like morphology SA‐β‐gal			0.25–10 μm for 3 days	Ewald *et al*. (2009)
(2c) DNA‐damaging drugs with complex effects							
actinomycin D DNA intercalator Inhibits transcription Can poison topoisomerases I and II, and, thus induce SSBs and DSBs	Normal human fibroblasts	Premature senescence	Growth inhibition	p53‐p21↑	G1 G2	0.04 mg mL^−1^ for 12 h	Robles and Adami (1998)
	Human mesenchymal stem cells	Premature senescence	Growth inhibition CS‐like morphology SA‐β‐Gal γ‐H2AX foci SASP	p53‐p21↑ p16↑		400 mm for 3–21 days	Minieri *et al*. (2015)
bleomycin Induces DNA breaks	Normal human fibroblasts	Premature senescence	Growth inhibition SA‐β‐gal	p53‐p21↑ p16↑	G1 G2	0.06 units mL^−1^ for 12–24 h	Robles and Adami (1998)
	A549 Rat primary type II cells C57BL/6J mice	Premature senescence	Growth inhibition CS‐like morphology SA‐β‐gal	p21↑		50 μg mL^−1^ for 120 h or 5 mg kg^−1^ day^−1^ for 7–21 days *in vivo*	Aoshiba *et al*. (2003)
	A549	Premature senescence	Growth inhibition CS‐like morphology SA‐β‐gal	p53‐p21↑	G2	50 mU mL^−1^ for 1–7 days siRNA for caveolin‐1 reduces SA‐β‐gal	Linge *et al*. (2007)
	BJ 293T	Premature senescence	CS‐like morphology SA‐β‐gal SASP			100 μg mL^−1^ for 24 h	Pazolli *et al*. (2012)
	C57BL/6J mice	Premature senescence	γH2AX p‐53BP1 SASP	p21↑ p‐ATM/ATR↑ p‐p38↑		2.5 mg kg^−1^ day^−1^ for 7–21 days *in vivo*	Aoshiba *et al*. (2013)
temozolomide DNA‐alkylating agent Alkylates/methylates DNA Induces DNA damage	U‐87 MG	Premature senescence	Growth inhibition CS‐like morphology SA‐β‐Gal	p53↑^▼^, p21↑	G2	100 μm for 3 h the gradual appearance of hyperploid cells	Hirose *et al*. (2001)
	Me4405 IR3 Mel‐CV MM200 SK‐mel‐28 Mel‐FH	Premature senescence	Growth inhibition CS‐like morphology SA‐β‐Gal	p53↑^▼^, p21↑	G2	25–100 μm for 72 h the gradual appearance of hyperploid cells	Mhaidat *et al*. (2007)
(3) Epigenetic modifiers							
5‐aza‐2′‐deoxycytidine Inhibitor of DNA methyltransferases Induces DSBs	MDAH041	Premature senescence	CS‐like morphology SA‐β‐gal	p16↑	S	1 μm for 6 days	Vogt *et al*. (1998)
	HepG2 NMRI mice	Premature senescence	Growth inhibition CS‐like morphology SA‐β‐gal γ‐H2AX accumulation of H3K9me3 SASP	p53^▼^ p16↑		20–50 μm for 96 h or 0.8 mg kg^−1^ day^−1^ for 3 days *in vivo*	Venturelli *et al*. (2013)
	U2OS	Premature senescence	Growth inhibition CS‐like morphology SA‐β‐Gal	p53↑ p21↑ p16 ↑		5–10 μm for 2–4 days	Widodo *et al*. (2007)
	H28	Premature senescence	Growth inhibition CS‐like morphology SA‐β‐Gal	p21↑ p27↑ ATM, ATR↑		0.1–10 μm for 2–6 days	Amatori *et al*. (2011)
sodium butyrate Class I and II histone deacetylase (HDAC) inhibitor	WI38	Senescence‐like state	Growth inhibition CS‐like morphology SA‐β‐gal	p‐ Rb ↓	G1	0.5 mm for ~20 days	Ogryzko *et al*. (1996)
	NIH3T3	Senescence‐like state	CS‐like morphology	p21↑	G1	5–10 mm for 48 h activation of p21 expression may be both p53‐dependent and p53‐independent	Xiao *et al*. (1997)
	HHUA Hec1‐A SKOV‐3 HeLa SiHa	Senescence‐like state	SA‐β‐gal	p21↑ p‐Rb↓	G1 G2	1–4 mm for 2–5 days activation of p21 expression may be both p53‐dependent and p53‐independent	Terao *et al*. (2001)
	WI‐38	Senescence‐like state	CS‐like morphology SA‐β‐gal	p21↑		4 mM for 24 h or 0.5 mM for 14 days	Place *et al*. (2005)
	E1A + Ras‐transfected rat and mouse embryonic fibroblasts	Premature senescence	γ‐H2AX	p21↑^▼^ p16↑	G1	4 mM for 24–72 h DDR without detectable DNA damage	Abramova *et al*. (2006), Pospelova *et al*. (2009)
	BJ 293T	Premature senescence	CS‐like morphology SASP	p53‐ and RB‐independent		4 mM for 3‐6 days SASP dependent upon ATM and NF‐κB	Pazolli *et al*. (2012)
trichostatin A Class I and II HDAC inhibitor	WI38	Senescence‐like state	Growth inhibition		G1 phase	10 ng mL^−1^ for ~30 days	Ogryzko *et al*. (1996)
	WI‐38	Senescence‐like state	CS‐like morphology SA‐β‐gal	p21↑		2 μm for 24–72 h or 0.5 μm for 9 days	Place *et al*. (2005)
	BJ 293T	Premature senescence	CS‐like morphology SA‐β‐gal SASP	p53‐ and RB‐independent		1 mM for 3 days lack of DNA damage	Pazolli *et al*. (2012)
	A549	Premature senescence	Growth inhibition CS‐like morphology	p21↑ p27↑	G1 G2	0.5–1.0 μm for 48 h	Zhao *et al*. (2010)
MS‐275 Class I HDAC inhibitor	Mesenchymal stem cells	Premature senescence	SA‐β‐gal	p16↑	Predominantly G2	1 μm for 72 h	Di Bernardo *et al*. (2009)
SAHA (vorinostat) Class I and II HDAC inhibitor	HCT116	Premature senescence	Growth inhibition CS‐like morphology SA‐β‐Gal	p53‐ and p21‐independent	G1 G2	0.4–1 μm for 5 days induced polyploid cells	Xu *et al*. (2005)
LBH589 (panobinostat) Class I and II HDAC inhibitor	B143 MG‐63 Saos‐2 SJSA U2OS human osteosarcoma cell lines	Premature senescence	Growth inhibition CS‐like morphology SA‐β‐Gal SASP	p53‐independent	G1	15 nm for 21 days aysor 2–10 mg kg^−1^ day^−1^ for 17 days *in vivo* (mice)	Cain *et al*. (2013)
4‐phenylbutyric acid Class I and IIa HDAC inhibitor	MCF7 HT1080	Premature senescence	Growth inhibition CS‐like morphology SA‐β‐Gal	Akt‐p21↑^▼^		200–500 μm for 6 days	Kim *et al*. (2012)
valproic acid Class I and IIa HDAC inhibitor	D283‐Med DAOY PFSK	Premature senescence	Growth inhibition CS‐like morphology SA‐β‐Gal	p21↑	G1	0.6–1 mm for 3–7 days or 400 mg kg^−1^ day^−1^ for 28 days *in vivo*	Li *et al*. (2005)
	Bel‐7402 Bel‐7404	Premature senescence	Growth inhibition CS‐like morphology SA‐β‐Gal	p21↑, pRb↓	G1	0.2–0.5 mm for 2–5 days	An *et al*. (2013)
curcumin and C646 p300 histone acetyltransferase inhibitors	TIG3	Senescence‐like state	SA‐β‐gal SAHF	p53‐, p21‐ and p16‐independent	G2	6–9 μm for 2–15 days Global H3, H4 hypoacetylation Lack of DNA damage	Prieur *et al*. (2011)
	HCT116 MCF 7 U2OS	Premature senescence	CS‐like morphology SA‐β‐gal	p21↑ p53‐independent	G2	10 μm (HCT116) or 15 μm (MCF 7) or 7.5 μm (U2OS) for 24 h	Mosieniak *et al*. (2012)
	CAF myofibroblasts	Premature senescence	CS‐like morphology SA‐β‐gal	p16↑^▼^ p53↑ p21↑		10 μm for 24 h	Hendrayani *et al*. (2013)
	VSMC endothelial cells derived from aorta	Premature senescence	Growth inhibition CS‐like morphology SA‐β‐gal SASP	p21↑ p16↑ p‐p53Ser15↑ p‐p38↑	G2	5–7.5 μm (VSMCs) and 2.5–5 μm (endothelial cells) for 3–7 days ROS‐ and ATM‐independent	Grabowska *et al*. (2015)
BRD4770 G9a histone methyltransferase inhibitor	PANC‐1	Premature senescence	Growth inhibition CS‐like morphology SA‐β‐Gal	p‐ATM↑	G2	10 μm for 24 h	Yuan *et al*. (2012)
(4) Inhibitors of telomerase activity							
SYUIQ‐5 Stabilizes G‐quadruplexes Induces TRF2 delocalization from telomeres	K562 SW620	Premature senescence	Growth inhibition SA‐β‐Gal	p16↑ p21↑ p27↑		0.2–0.4 μm for 16–35 days	Zhou *et al*. (2006)
BMVC4 Stabilizes G‐quadruplexes	H1299 MCF7 HeLa VA13 SaoS2 U2OS	Premature senescence	Growth inhibition SA‐β‐Gal SAHF (H3K9me3) γ‐H2AX foci	p‐ATM↑^▼^ p‐Rb↓	S	1–10 μm for 9–12 days	Huang *et al*. (2012)
pyridostatin Stabilizes G‐quadruplexes	HT1080	Premature senescence	Growth inhibition SA‐β‐Gal			0.3–40 μm for 8 days	Muller *et al*. (2012)
compound 115405 G‐quadruplex ligand	A549	Premature senescence	Growth inhibition CS‐like morphology SA‐β‐Gal			0.4 μm for ~40 days	Riou *et al*. (2002)
perylene derivatives PM2 and PIPER Induce G‐quadruplex formation from both telomeric DNA and hTERT promoter region	A549	Premature senescence	Growth inhibition CS‐like morphology SA‐β‐Gal			0.4–0.8 μm for 24–78 days	Taka *et al*. (2013)
harmine β‐carboline alkaloid	MCF7	Premature senescence	Growth inhibition CS‐like morphology SA‐β‐Gal γ‐H2AX	p53‐p21↑		20–30 μm for 48–96 h	Zhao and Wink (2013)
indole derivatives (indole‐3‐carbinol (I3C) and indoxyl sulfate) Phytochemicals that downregulate hTERT expression	MCF7	Premature senescence	Growth inhibition SA‐β‐Gal		G1	200 μm for 48 h	Marconett *et al*. (2011)
	HK‐2 CRF rats	Premature senescence	Growth inhibition SA‐β‐Gal	p53↑ p‐p53Ser15↑		250 μm for 48–120 h or 4 g kg^−1^ for 16 weeks *in vivo*	Shimizu *et al*. (2010)
	HK‐2	Premature senescence	Growth inhibition SA‐β‐Gal	NF‐κB↑^▼^		250 μm for 48–72 h ROS↑	Shimizu *et al*. (2011)
BIBR1532 Non‐nucleosidic TERT inhibitor	NCI‐H460	Senescence‐like phenotype	Growth inhibition CS‐like morphology SA‐β‐Gal			10 mm for 130 days	Damm *et al*. (2001)
azidothymidine (AZT) Reverse transcriptase inhibitor Inhibits telomerase activity	MCF‐7	Senescence‐like arrest	Growth inhibition SA‐β‐Gal			20 and 70 μm for ~ 50–60 population doublings (PD)	Ji *et al*. (2005)
	HTLV‐I ATL patients	Premature senescence	Growth inhibition SA‐β‐Gal	p53↑^▼^ p21↑ p27↑		50 μm for 18 weeks *in vivo* (ATL patients) the Jurkat T‐cell line, treated under the same conditions, did not enter growth arrest	Datta *et al*. (2006)
	MASC C57BL/6 mice	Premature senescence	Growth inhibition SA‐β‐Gal			30 μm for 48 h or 100 mg kg^−1^ day^−1^ for 2 weeks *in vivo*	Demir and Laywell (2015)
(5) cyclin‐dependent kinase (CDK) inhibitors							
palbociclib (PD‐0332991) CDK4 and CDK6 inhibitors	HT1080 MEL10 RPE	Premature senescence	Growth inhibition CS‐like morphology SA‐β‐Gal	mTOR^▼^ pRb↓		0.5 μm for 3–7 days	Leontieva and Blagosklonny (2013)
	MEL 10 RPE	Premature senescence	Growth inhibition CS‐like morphology SA‐β‐Gal	mTOR, MEK^▼^		1 μm for 5 days	Leontieva *et al*. (2013)
	12 sarcoma cell lines generated directly from patient samples	Premature senescence	Growth inhibition CS‐like morphology SA‐β‐Gal 53BP1 foci	pRb↓	G1	9–27 μm for 2–4 days or 100 mg kg^−1^ day^−1^ for 3 weeks *in vivo* (mice)	Perez *et al*. (2015)
	MCF7	Premature senescence	Growth inhibition SA‐β‐Gal		G1	1 μm for 5–7 days, 0.6 or 3.0 mg kg^−1^ day for 10 cycles each consisting of 3 weeks *in vivo* (dogs)	Hu *et al*. (2015)
	1205Lu WM983 WM983BR WM451Lu WM239A WM3918	Premature senescence	Growth inhibition CS‐like morphology SA‐β‐Gal SASP SAHF	pRb↓ mTOR↓	G1	1 μm for 8 days or 90 mg kg^−1^ day for 14 days *in vivo* (mice)	Yoshida *et al*. (2016)
roscovitinE (seliciclib) CDK2, CDK7, and CDK9 inhibitors	RTE MDCK WT 9‐7	Premature senescence	Growth inhibition CS‐like morphology SA‐β‐Gal	p53‐p21↑		1–10 μg mL^−1^ for 24 h	Park *et al*. (2009)
ribociclib (LEE011) CDK4 and CDK6 inhibitors	Human neuroblastoma‐derived cells	Premature senescence	Growth inhibition SA‐β‐Gal	p‐Rb↓	G1	500 nm for 6 days or 200 mg kg^−1^ for 21 days *in vivo* (mice)	Rader *et al*. (2013)
(6) p53 activators							
nutlin‐3a Inhibits MDM2 binding to p53	MEF oncogenically transformed MEF murine fibrosarcoma cell lines	Premature senescence	Growth inhibition CS‐like morphology SA‐β‐Gal	p53↑^▼^		5 or 10 μm for 1–7 days no apoptosis was observed	Efeyan *et al*. (2007)
	MCF‐7	Premature senescence	Growth inhibition CS‐like morphology SA‐β‐Gal	p53^▼^	G1 G2	10 μm for 5 days	Huang *et al*. (2011)
	MyLa2000 Mac1 Mac2a	Premature senescence	Growth inhibition SA‐β‐Gal	p53↑^▼^ p21↑	G1	2.5–10 μm for 24–72 h induces apoptosis	Manfe *et al*. (2012)
FL118 Camptothecin analogue Induces proteasomal degradation of MdmX	HCT116 HCT8	Premature senescence	Growth inhibition CS‐like morphology SA‐β‐Gal	p53↑^▼^ p21↑		10 nm for 3 days	Ling *et al*. (2014)
(7) activators of protein kinase C (PKC)							
TPA/PMA (12‐O‐tetradecanoylphorbol‐13‐acetate/(phorbol‐12‐myristate‐13‐acetate) Activates PKC Induces DNA damage	D04 D08 MM127 MM455	Premature senescence	Growth inhibition SA‐β‐Gal	p21↑ ERK↑ p‐Rb↓	G1	0.1–1 μg mL^−1^ for 24 h telomerase was selectively repressed; normal human fibroblasts were resistant to treatment	Cozzi *et al*. (2006)
	H358 H441 H322	Premature senescence	Growth inhibition CS‐like morphology SA‐β‐Gal	p21↑^▼^ pRb↓	G2	100 nm for 30 min reduced telomerase activity	Oliva *et al*. (2008)
	SK‐MEL‐5 MCF7 COLO‐205	Senescence‐like arrest	Growth inhibition SA‐β‐Gal	p21↑ ERK1/2↑ p‐Rb↓	G2	10‐1000 ng mL^−1^ for 24 h	Mason *et al*. (2010)
PEP005 (ingenol‐3‐angelate) Activates PKC	D04 D08 MM127 MM455	Premature senescence	Growth inhibition SA‐β‐Gal	p21↑ ERK↑	G1	0.2–1 μg mL^−1^ for 24 h telomerase was selectively repressed normal human fibroblasts were resistant to treatment	Cozzi *et al*. (2006)
PEP008 (20‐O‐acetyl‐ingenol‐3‐angelate)	SK‐MEL‐5 MCF7 COLO‐205	Senescence‐like arrest	Growth inhibition SA‐β‐Gal	p21↑ ERK1↑ p‐Rb ↓	G2	10–1000 ng mL^−1^ for 24 h or 5–6 days	Mason *et al*. (2010)
(8) ROS inducers							
hydrogen peroxide (H_2_O_2_)	F65	Senescence‐like arrest	CS‐like morphology SA‐β‐Gal			200 μm for 2 h	Chen and Ames (1994)
	IMR‐90	Senescence‐like arrest	CS‐like morphology SA‐β‐Gal	p53‐p21↑ p‐Rb↓	G1	300 μm for 2 h	Chen *et al*. (1998)
	IMR‐90	Senescence‐like arrest	CS‐like morphology SA‐β‐Gal	p‐p38↑^▼^ p‐Rb↓		150 μm for 2 h	Frippiat *et al*. (2002)
	2BS	Premature senescence	CS‐like morphology SA‐β‐Gal	p53‐p21↑	G1	10 μm for 3 weeks accumulation of DNA damage accelerated telomere shortening	Duan *et al*. (2005)
	A549	Premature senescence	CS‐like morphology SA‐β‐Gal	p53‐p21↑ p‐Rb↓		100 μm for 2 h	Yoshizaki *et al*. (2009)
	Primary human keratinocytes	Premature senescence	CS‐like morphology SA‐β‐Gal	p53‐p21↑		50 μm for 2 h	Ido *et al*. (2012)
	HUVEC	Premature senescence	CS‐like morphology SA‐β‐Gal SASP	p53‐p21↑		100 μm for 1 h	Suzuki *et al*. (2013)
	hMESCs	Premature senescence	CS‐like morphology SA‐β‐Gal γH2A.X and p‐53BP1 foci	p‐p38↑^▼^ p53‐p21↑ p‐Rb↓	G1	200 μm for 1 h	Burova *et al*. (2013), Borodkina *et al*. (2014)
	WI‐38 IMR‐90 LF1 HCA2	Premature senescence	SA‐β‐Gal	p21↑^▼^ p‐Rb↓		500 μm for 2 h	Gorbunova *et al*. (2002)
	IMR‐90	Premature senescence	CS‐like morphology SA‐β‐Gal	p21↑ p‐ERK↑ p‐Akt↑		150 μm for 2 h caveolin 1↑	Chretien *et al*. (2008)
	IMR‐90	Premature senescence	SA‐β‐Gal	p‐p38↑^▼^ p21↑		200 μm for 2 h	Zdanov *et al*. (2006)
	HUVEC	Premature senescence	CS‐like morphology SA‐β‐Gal	p53↑		100 μm for 1 h	Ota *et al*. (2008)
	MRC‐5	Premature senescence					von Zglinicki *et al*. (2000)
* tert*‐butyl hydroperoxide (tBHP)	WI‐38	Premature senescence	CS‐like morphology SA‐β‐Gal	p21↑ p‐Rb↓		5 × 30 μm for 1 h day^−1^	Dumont *et al*. (2000)
	HUVEC	Premature senescence	CS‐like morphology SA‐β‐Gal			100 μm for 2 days	Kurz *et al*. (2004)
	WI‐38	Premature senescence	CS‐like morphology SA‐β‐Gal SASP			5 × 30 μm for 1 h day^−1^	Pascal *et al*. (2007)
	WI‐38	Premature senescence	CS‐like morphology SA‐β‐Gal	p21↑ p16↑	G1	4 × 100 μm for 1 h per every two doubling	Chen *et al*. (2008)
	Human mesangial cells	Premature senescence	CS‐like morphology SA‐β‐Gal	JAK2‐STAT↑^▼^	G1	30 μm for 1 h	Zhou *et al*. (2013)
phenyl‐2‐pyridyl ketoxime (PPKO)	Primary human fibroblasts were isolated from newborn foreskins	Senescence‐like arrest	CS‐like morphology SA‐β‐Gal	p53‐p21↑ p16↑ ERK1/ERK2↑ ROS‐ and NO↑‐dependent	G2	1 mm for 3 days	Yang *et al*. (2016)
phenylaminonaphthoquinones (Q7 and Q9)	T24	Senescence‐like arrest	CS‐like morphology SA‐β‐Gal	MAPK^▼^ p53‐p21↑ p27↑	G2	4 μm for 1–3 days alone or with 1 mM ascorbate	Felipe *et al*. (2013)
paraquat	TIG‐7	Premature senescence	CS‐like morphology SA‐β‐Gal			100 μm for 4 days	Joguchi *et al*. (2004)
	BALB/c mice	Premature senescence	SA‐β‐Gal			25 mg kg^−1^ for 3 days (intraperitoneal injection)	Ota *et al*. (2008)
	BJ	Premature senescence	CS‐like morphology SA‐β‐Gal	p53↑ p16↑		350 μm for 16 h thioredoxin 1↑	Young *et al*. (2010)

a *Cell lines*: 1205Lu, human lung melanoma cells; 2BS, human embryonic lung fibroblasts; A2780, human ovarian carcinoma cells; A549, human lung adenocarcinoma epithelial cells; AsPC1, human pancreas adenocarcinoma cells; B143, human osteosarcoma cells; Bel‐7402, human hepatocellular carcinoma cells; Bel‐7404, human hepatocellular carcinoma cells; BJ, normal human foreskin fibroblasts; CAF, cancer‐associated fibroblasts; CAL27, human oral adenosquamous carcinoma cells; CNE1, human nasopharyngeal carcinoma cells; D283‐Med, human medulloblastoma cells; DAOY, human cerebellar medulloblastoma cells; DU145, human prostate carcinoma cells; F65, human foreskin fibroblasts; H1299, human lung carcinoma cells; H28, human mesothelioma cells; H9c2, rat cardiomyoblast cells; HCA2, normal human foreskin fibroblasts; HCT116, human colorectal carcinoma cells; HCT8, human ileocecal colorectal adenocarcinoma cells; Hec1‐A, human uterus/endometrium adenocarcinoma cells; HeLa, human cervix adenocarcinoma cells; HepG2, human hepatocellular carcinoma cells; HFF, human foreskin normal fibroblasts; HHUA, human endometrial cells; HK‐2, human renal proximal tubule cells; hMESCs, human endometrium‐derived mesenchymal stem cells; HT1080, human connective tissue fibrosarcoma cells; HTLV‐I, human T‐cell leukemia virus type I (HTLV‐I)‐infected cells; HUVEC, human umbilical vein endothelial cells; IMR‐90, human fetal lung fibroblasts; Jurkat, human T‐cell leukemia cells; K562, human bone marrow myelogenous leukemia lymphoblasts; LF1, human embryonic lung fibroblasts; LNCaP, human prostate carcinoma cells; LS174T, human colorectal adenocarcinoma cells; Mac1, human cutaneous T‐cell lymphoma (CTCL); Mac2a, human cutaneous T‐cell lymphoma (CTCL); MASC, mouse multipotent astrocytic stem cell; McA‐RH7777, rat hepatoma cells; MCF10, human breast fibrocystic cells; MCF7, human breast adenocarcinoma cells; MDAH041, derivate from the fibroblasts of a patient with Li–Fraumeni syndrome; MDCK, canine epithelial kidney cells; MEF, mouse embryonic fibroblasts; MEL10 (SK‐MEL‐147), human melanoma cells; MG‐63, human osteosarcoma cells; MRC‐5, human lung fibroblast; MyLa2000, human cutaneous T‐cell lymphoma (CTCL); NCI‐H460, human lung carcinoma cells; NIH3T3, mouse embryo fibroblasts; PANC‐1, human pancreatic carcinoma cells; PC‐3, human prostate cancer cells; PFSK‐1, human neuroectodermal cells derived from cerebral brain tumor; REF52, rat embryonic fibroblasts; RPE, human retinal pigment epithelial cells; RTE, rat tracheal epithelial cells; Saos‐2, human osteosarcoma cells; SiHa, human cervix squamous cell carcinoma cells; SJSA‐1, human osteosarcoma cells; SKN‐SH, human neuroblastoma cells; SKOV‐3, human ovary adenocarcinoma cells; SW620, human colon cancer cells; T24, human bladder carcinoma cells; TIG‐3, human embryonic lung fibroblasts; TIG‐7, human embryonic lung fibroblasts; U2OS, human osteosarcoma cells; U‐87 MG, human glioblastoma, astrocytoma cells; UM‐SCC1, human squamous carcinoma of the oral cavity cells; UM‐SCC14A, human squamous carcinoma of the oral cavity cells; VA‐13, human lung fibroblasts; VSMC, vascular smooth muscle cells; WI38, human lung fibroblasts; WM239A, human melanoma cells; WM3918, human melanoma cells; WM451Lu, human melanoma cells; WM983, human melanoma cells; WM983BR, human melanoma cells; WT 9‐7, human cells from a patient with autosomal‐dominant polycystic kidney disease (ADPKD).

b *Abbreviations*: CS, cellular senescence; DSBs, double‐stranded DNA breaks; SA‐β‐gal, senescence‐associated β‐galactosidase; SAHF, senescence‐associated heterochromatin foci; SASP, senescence‐associated secretory phenotype; SSBs, single‐stranded DNA breaks.

c *Symbols*: ↑, increased activity/expression reported; ↓, decreased activity/expression reported; ^▼^, involvement of the protein/pathway was verified by gene(s) knockout or knockdown, inhibitory analysis, and/or using cell lines carrying inactivating mutations.

The table highlights the fact that cancer cells can undergo cellular senescence *in vitro* just as well as their normal nontransformed counterparts. It is apparent that there is no senescence marker or pathway unique to normal or cancer cells. In most cases, increased SA‐β‐gal, morphological changes, and persistent DDR foci were recorded. SAHF were found in only a few cases (aphidicolin, etoposide, palbociclib, and epigenetic modifiers). SASP was also noted only in some cases; however, this is likely because SASP is not commonly analyzed as a senescence biomarker. Apparently, an implicit consensus was established that the demonstration of SA‐β‐gal, morphological changes, and persistent DDR is sufficient to document a cellular senescence‐like state. It is notable that authors designated these phenotypes as a state of premature senescence or senescence‐like cell cycle arrest, regardless of the set of biomarkers observed in each case.

Extremely prolonged drug exposure (from hours to days) was typically required to induce cellular senescence, as is evidenced by the table. In marginal situations, as in the case of aphidicolin‐induced cell cycle arrest, the full set of senescence biomarkers (SA‐β‐gal, cell enlargement, SAHF, and DDR foci) was maintained, while the drug was present in the culture medium and lost upon drug removal (Maya‐Mendoza *et al*., 2014). The requirement for prolonged incubation time was found for all groups of chemical compounds analyzed; however, the mechanism of senescence development appeared to differ among these groups. Whereas replication stress inducers, different DNA‐damaging agents, and telomerase inhibitors likely generate a persistent DDR following prolonged introduction of a small number of DNA lesions or telomere uncapping, long‐term incubation with epigenetic modifiers likely causes transcriptional activation of repressed loci (particularly INK4A, which encodes p16 CDK inhibitor). This hypothesis is supported by the fact that, in contrast to DNA damage‐induced cellular senescence, which depends on p21 CDK inhibitor, epigenetically induced senescence is mostly dependent on p16. This characterizes epigenetically induced senescence as ‘causeless’—epigenetic modifiers directly activate molecular pathways maintaining the cellular senescence state without generating any cell stress. In this regard, senescence induced by epigenetic modifiers can resemble developmentally programmed or organismal aging‐associated cellular senescence, while replication stress‐ and DNA damage‐induced senescence are examples of stress‐induced premature senescence states.

It follows from the table that replication stress‐ and DNA damage‐induced cellular senescence mostly depend on the p53‐p21 pathway. The same is basically true for cellular senescence induced by physical stressors such as ionizing radiation (IR) and ultraviolet (UV) (Latonen *et al*., 2001; Suzuki *et al*., 2006). It is well known that IR as well as UV can stimulate senescence in a variety of normal and cancer cell lines (Chainiaux *et al*., 2002; Meng *et al*., 2003; Jones *et al*., 2005; Jee *et al*., 2009). Mechanistically, this type of cellular senescence mostly depends on DNA damage induced by these stressors; this links IR and UV to chemical DNA‐damaging agents. Moreover, IR and UV, along with most of the DNA‐damaging agents presented in the table, induce apoptosis rather than senescence when used at higher doses. These observations further emphasize the relationship between apoptosis and senescence. Accordingly, these cell stress response pathways may operate either as alternatives or as supplement to each other. While prominent (but short term) DNA damage induces apoptosis, prolonged mild DNA damage activates cellular senescence. The p53 transcription factor emerges as a master regulator controlling these cell fate decisions (Purvis *et al*., 2012).

## Funding

This work was supported by a Russian Science Foundation [grant number 14‐24‐00022].

## Conflict of interest

None declared.
